# Reducing the Risk of Pre-Eclampsia in Women with Polycystic Ovary Syndrome Using a Combination of Pregnancy Screening, Lifestyle, and Medical Management Strategies

**DOI:** 10.3390/jcm13061774

**Published:** 2024-03-20

**Authors:** Jim Parker, Claire Louise O’Brien, Christabelle Yeoh, Felice L. Gersh, Shaun Brennecke

**Affiliations:** 1 School of Medicine, University of Wollongong, Wollongong 2522, Australia; 2 Faculty of Science and Technology, University of Canberra, Canberra 2617, Australia; claire.obrien@canberra.edu.au; 3 Next Practice Genbiome, 2/2 New McLean Street, Edgecliff 2027, Australia; christabelle.yeoh@nextpracticehealth.com; 4 College of Medicine, University of Arizona, Tucson, AZ 85004, USA; felicelgersh@yahoo.com; 5 Department of Maternal-Fetal Medicine, Pregnancy Research Centre, The Royal Women’s Hospital, Melbourne 3052, Australia; s.brennecke@unimelb.edu.au; 6 Department of Obstetrics and Gynaecology, The University of Melbourne, Melbourne 3052, Australia

**Keywords:** PCOS, pre-eclampsia, pregnancy, lifestyle, nutrition, placenta, pathophysiology, angiogenic ratio, screening

## Abstract

Polycystic ovary syndrome (PCOS) is a multisystem disorder that presents with a variety of phenotypes involving metabolic, endocrine, reproductive, and psychological symptoms and signs. Women with PCOS are at increased risk of pregnancy complications including implantation failure, miscarriage, gestational diabetes, fetal growth restriction, preterm labor, and pre-eclampsia (PE). This may be attributed to the presence of specific susceptibility features associated with PCOS before and during pregnancy, such as chronic systemic inflammation, insulin resistance (IR), and hyperandrogenism, all of which have been associated with an increased risk of pregnancy complications. Many of the features of PCOS are reversible following lifestyle interventions such as diet and exercise, and pregnant women following a healthy lifestyle have been found to have a lower risk of complications, including PE. This narrative synthesis summarizes the evidence investigating the risk of PE and the role of nutritional factors in women with PCOS. The findings suggest that the beneficial aspects of lifestyle management of PCOS, as recommended in the evidence-based international guidelines, extend to improved pregnancy outcomes. Identifying high-risk women with PCOS will allow targeted interventions, early-pregnancy screening, and increased surveillance for PE. Women with PCOS should be included in risk assessment algorithms for PE.

## 1. Introduction

Polycystic ovary syndrome is a systemic metabolic and endocrine disorder that results from the disturbance of adaptive interdependent homeostatic survival networks (metabolic, immune, and neuroendocrine) [1,2,3,4]. Although a wide variety of theories have been proposed to explain the pathogenesis of PCOS [5,6,7,8,9,10], recent evolutionary models have provided a unified framework [1,2]. Evolutionary models characterize PCOS as an evolutionary mismatch disorder that arises from an interaction between genetic and environmental factors [1,2]. Rapid cultural changes in the contemporary environment have outpaced genetic adaptation and resulted in a mismatch between modern dietary and environmental exposures and behaviors, and selective metabolic and reproductive traits [1]. As a result, adaptive physiological survival pathways that result in the activation of inflammation, variation in insulin sensitivity, preferential abdominal fat accumulation, and the downregulation of reproduction, become pathological following exposure to lifestyle and environmental factors [1,2]. The development of chronic systemic inflammation, IR, and hyperandrogenism predispose women with PCOS to a range of chronic diseases and pregnancy complications [11,12,13,14,15].

Polycystic ovary syndrome affects 10–13% of reproductive-age women and can present with a wide range of symptoms, including menstrual irregularity, hirsutism, acne, alopecia, anxiety, depression, and subfertility, resulting in reduced quality of life [16]. PCOS can be a progressive metabolic condition that leads to obesity, hypertension, dyslipidemia, type 2 diabetes, metabolic syndrome, metabolic-associated hepatic steatosis, chronic kidney disease, cardiovascular disease, and cancer [1,10,13,17]. The population-attributable risk of PCOS to type 2 diabetes is 19–28%, and the combined impact of PCOS makes a significant contribution to the chronic disease epidemic [13]. Multiple systematic reviews of large population-based studies over the past 40 years have reported a significantly increased risk of PE in women with PCOS [18,19,20,21,22,23].

It has long been appreciated that normal placental development requires a complex network of bidirectional communication signals (cytokines, metabolites, hormones, exosomes) between embryo-derived cells (trophoblasts, macrophages) and maternally derived cells (endometrial gland epithelium, stromal, macrophage, natural killer, dendritic, and T cells) [24,25]. Decades of epidemiological research has identified over 70 maternal risk factors that are associated with the development of PE [25,26]. This has resulted in a greater awareness of the possible role of pre-existing maternal pathophysiological features on the development of abnormal placentation and related complications, such as PE [24]. Although there may be differences in the pathogenesis of early-onset (<34 weeks’ gestation) and late-onset PE, the pathophysiology and maternal/fetal consequences can be similar [24]. Pre-existing maternal pathological features such as chronic systemic inflammation [27], insulin resistance [28], and hyperandrogenemia [29], as occur in women with PCOS [3], may alter normal placental development, metabolism, and physiology, at all stages of pregnancy [30,31].

## 2. Scope and Methodology

This review aims to provide recommendations for the screening and management of women with PCOS to reduce their risk of PE in pregnancy. This review is an overview and synthesis of evidence from systematic reviews, observational studies, and molecular research into the risk of PE in women with PCOS. It includes a summary of systematic reviews examining the evidence for increased risk of PE in women with PCOS (Section 4), a review of evidence for the role of nutritional factors in the pathogenesis of PE (Section 5), and a summary of the possible mechanisms (Section 6). This includes an overview of the role of IR (Section 6.3), chronic systemic inflammation (Section 6.4), hyperandrogenism (Section 6.5), and recent evidence on the role of the maternal microbiome (Section 6.6) in the pathophysiology of PE. Readers are directed to recent state-of-the-art in-depth reviews where appropriate.

These sections provide the background to recommendations for multivariable screening in early pregnancy, medical and lifestyle management, and second-trimester assessments using angiogenic factors in women with PCOS. The multivariable involves a combination of the maternal mean arterial blood pressure, ultrasound assessment of uterine artery pulsatility index (UtAPI), and serum placental growth factor (PlGF), and is performed between 11 and 13 weeks and at 6 days of gestation. Medical management with aspirin is discussed, and lifestyle management refers to diet, body weight management, physical activity, stress, sleep, and social support. The angiogenic factors discussed in this review refer to soluble fms-like tyrosine kinase-1 (sFlt-1) and PlGF. The list of bibliographic references was based on PubMed, MEDLINE, Scopus, and Cochrane databases. Databases were searched until January 2024 and only articles in English were considered. This review is a narrative synthesis and no attempt was made to perform a systematic review or to combine data due to the heterogeneity of the studies included.

## 3. Hypertensive Disorders of Pregnancy and PE

Hypertensive disorders of pregnancy (HDPs) (defined as chronic hypertension, gestational hypertension, pre-eclampsia–eclampsia, and chronic hypertension with superimposed pre-eclampsia) are a leading cause of maternal–fetal morbidity and mortality worldwide [32,33]. Pre-eclampsia affects 3–5% of pregnancies and is responsible for 76,000 maternal and 500,000 fetal/neonatal deaths every year [32,34]. The definition of PE has evolved over time, in line with research developments into the underlying pathophysiology [35]. This has resulted in an increased awareness of factors involved in the prevention, prediction, diagnosis, treatment, and long-term consequences of PE. It has been estimated that millions of women are at risk of chronic health problems (neurodevelopmental, metabolic, cardiac) due to having previously experienced PE [36,37,38].

The International Society for the Study of Hypertension in Pregnancy (ISSHP) has defined PE as new-onset gestational hypertension at or after 20 weeks’ gestation accompanied by proteinuria, maternal organ involvement, or uteroplacental dysfunction [33]. The pathophysiology of PE is characterized by placental malperfusion that results in syncytiotrophoblast stress and the release of soluble factors (pro-inflammatory cytokines, exosomes, extracellular vesicles, transcription factors, hormones, and anti-angiogenic factors), which cause maternal vascular endothelial injury, resulting in hypertension and multi-organ involvement [24,25,39]. More recently, circulating angiogenic factors such as sFlt-1 and PlGF have been identified as markers of placental health and added to the diagnostic criteria in some countries [35]. In addition, advances in genetics, epigenetics, transcriptomics, metabolomics, artificial intelligence, organoid cultures of the endometrium and trophoblasts, and stem cell research have progressed our understanding of normal and pathological placentation [24,25].

## 4. Evidence for the Increased Risk of PE in Women with PCOS and the Inclusion of PCOS in Clinical Practice Guidelines

### 4.1. Summary of Systematic Reviews

Many observational studies, systematic reviews, and meta-analyses have reported an increased risk of pregnancy complications in women with PCOS over the past 40 years. These include miscarriage, gestational diabetes mellitus (GDM), intrauterine growth restriction, preterm birth, low birth weight, gestational hypertension, and PE [18,19,20,21,22]. In total, eight systematic reviews published between 2006 and 2023 reported an increased relative risk of PE in women with PCOS of between 1.87 and 4.23 (Table 1) [18,19,20,21,22,40,41,42]. The most recent 2023 meta-analysis included 36 studies that compared rates of PE in women with and without PCOS [42]. A pooled meta-analysis of 34 studies involving women not taking metformin showed a significantly increased risk of PE in women with PCOS (OR: 2.35, 95% CI: 1.93–2.86). A subgroup analysis of high-quality studies, after the removal of low- and medium-quality studies, showed a significantly higher risk of PE in women with PCOS (OR: 3.05, 95% CI: 1.20–7.8). A subgroup analysis of seven body mass index (BMI)-matched studies showed that women with PCOS retained an increased risk of developing PE (OR: 2.39, 95% CI: 1.14–4.99) [42]. These findings are in agreement with two previous meta-analyses that showed a higher prevalence of PE in BMI-matched women with and without PCOS [40,41]. Overall, these data suggest that women with PCOS have an increased risk of PE that is independent of their BMI.

### 4.2. Risk of PE according to the United States National Inpatient Database

In addition, a large study from the United States National Inpatient Database of 71,436,308 weighted hospitalizations for deliveries analyzed 195,675 women with PCOS for their risk of pregnancy complications [23]. Women with PCOS had a higher risk of PE, eclampsia, peripartum cardiomyopathy, and heart failure during delivery hospitalizations. The risk of developing PE was significantly increased in women with PCOS after adjustment for age, race, demographic variables, and comorbidities, including BMI (OR: 1.56, 95% CI: 1.54–1.59). In addition, delivery hospitalizations were associated with increased lengths and costs of hospitalization in women with PCOS [23]. Despite this large body of evidence, PCOS is not generally recognized as a risk factor for pregnancy complications and PE and is not included in national or international risk assessment models.

### 4.3. PCOS and Clinical Practice Guidelines

There is an international consensus that women should be assessed for risk factors associated with PE in early pregnancy [26,33,43]. There is ongoing debate regarding which risk factors to include and the type of measures that should be part of risk assessment strategies [43]. Clinical practice guidelines (CPGs) recommend a combination of predictive assessments that include clinical risk factors, biophysical markers such as MAP and UtAPI, and biochemical markers such as pregnancy-associated plasma protein A (PAPP-A) and/or PlGF [44,45]. Various combinations of these measures are advised by different national bodies and professional societies [32,43,46]. Recent reviews and commentaries have highlighted the fact that PE risk factors in the current CPGs are poorly aligned with the evidence [26,47]. A recent review by an expert working group identified PCOS as a probable risk factor for the development of PE, having a similar relative risk and level of evidence to many other risks that are currently included in risk assessment models [26]. None of the current CPGs include PCOS as a risk factor. More recent studies add further weight to this evidence and support the need for a review of the strategies advocated in the CPGs [23].

The international evidence-based guidelines for the assessment and management of PCOS recommend the screening, monitoring, and management of risk profiles in women with PCOS, pre-conception, during pregnancy, and postpartum, in accordance with the recommendations for the general population [16,48]. These recommendations include the assessment of their blood glucose, body weight, blood pressure, smoking habits, alcohol consumption, diet, exercise, sleep, and emotional health. There are no specific recommendations for the identification, screening, and management of women with PCOS in pregnancy.

## 5. Evidence for the Role of Nutritional Factors in the Pathophysiology of PE

### 5.1. Role of a Healthy Lifestyle and Diet in the Pathogenesis of PE

Maternal nutrition has been suspected to play a role in the pathogenesis of PE for over 100 years [49,50,51]. This “dietary” hypothesis was proposed by advocates in the United Kingdom (Theobald) and United States (Dieckmann) in the early 1900s [50]. One 1926 review summarized the literature up to this time on the possible pathological effects of dietary macronutrients and their metabolites (protein and urea, fat and ketosis, carbohydrate restriction and ketosis) in patients with PE [51]. Although dietary management was common (milk, bread, rice, eggs, and fruit, with salt restriction), it was noted that there were “no series of experiments in which the effect of various diets in PE has been deliberately tested” [51]. Nevertheless, it was noted that the incidence of eclampsia fell significantly during the First World War and Second World War and increased in the post-war years in Germany and the Netherlands, respectively, and this was attributed to dietary restriction during the war years [50]. In addition, there were numerous publications between 1922 and 1957 that reported differences in the rates of PE and eclampsia in indigenous versus European and urban populations (Algeria 1922, India 1938, Ceylon 1946, South Africa 1947, New Guinea 1949, Fiji 1950, Indonesia 1952, Belgian Congo 1956) [49,50]. Indigenous diets were noted to consist of “wholefoods” containing starchy root vegetables, leafy greens, home-pounded grains, fruit, and small quantities of milk, fish, and meat, if they were available. This was contrasted with European and urban diets that were high in refined grains (white rice and flour), sugar, and salt, and low in meat, milk, vegetables, and fruit [50].

According to the Global Burden of Disease study, a poor-quality diet is one of the leading risk factors for morbidity and mortality globally [52]. International guidelines for the assessment and management of PCOS have recommended that lifestyle interventions, such as interventions related to diet and exercise, should be discussed with all women diagnosed with PCOS [53]. More recent observational and intervention studies provide contemporary evidence to support the role of nutritional factors in PE [54]. Evidence-based summaries from systematic reviews, meta-analyses, and representative PE expert groups (such as PRECISE) recommend healthy maternal dietary patterns to reduce the risk of PE [54,55,56,57].

One recent comprehensive evidence-based expert review of nutritional factors that may protect or exacerbate the risk of PE by the PRECISE Conceptual Framework Working Group highlighted the importance of focusing on research related to healthy dietary patterns rather than single nutrients [55]. Nevertheless, they identified 25 nutritional factors in two umbrella reviews and twenty-two meta-analyses. Of these, 14 were found to be significantly associated with an increased incidence of PE. Healthy maternal diets containing fruits, vegetables, whole-grain foods, fish, and chicken, such as the Mediterranean and New Nordic diets, were associated with 22%-reduced odds of developing PE (OR: 0.78, 95% CI: 0.70–0.86) [57]. In contrast, maternal diets high in ultra-processed foods and added sugars increased the odds of developing PE by 28% (OR: 1.28, 95% CI: 1.15–1.42) [58]. Long-term longitudinal studies have shown that higher ultra-processed food intake in women is associated with increased cardiovascular risk and hypertension [59], as can occur in women with a history of PE [60]. These data support the recommendations of other expert reviews and the World Health Organization regarding promoting healthy maternal diets [55,61,62].

Prospective cohort studies and observational research have suggested that following a healthy lifestyle and diet prior to pregnancy is associated with a reduced risk of PE. One multicenter prospective SCOPE study enrolled 5628 apparently healthy nulliparous women with singleton pregnancies to examine the association of PCOS (354 women) with pregnancy complications, including PE [63]. The investigators reported that in this low-risk population, the proportion of women with PE was similar among women with PCOS to that among those without PCOS (5.9% vs. 6.7%; OR: 0.88, 95% CI: 0.56–1.4). The pregnant women with PCOS were following a healthier lifestyle, including increased fruit and vegetable intake, more frequent vigorous exercise, lower alcohol consumption, and lower rates of smoking [63]. An analysis of the data from the complete SCOPE cohort of 5628 women showed that lower intakes of fruit and higher intakes of fast food in the pre-conception period were associated with longer times to pregnancy [64]. Another recent large meta-analysis of 21 cohort studies showed that following a healthy lifestyle (a healthy diet and high physical activity) can also reduce the risk of developing GDM [65]. These authors highlighted the need for more randomized intervention trials. Taken together, these data suggest that nutritional factors may have an impact on fertilization, implantation, and placentation.

### 5.2. Future Research into the Role of Lifestyle Modification in Reducing the Risk of PE in Women with PCOS

Intervention trials currently underway should help to clarify the impact of lifestyle modification prior to pregnancy on in utero metabolic factors and neonatal outcomes [66]. Recent developments in endometrial organoid research should provide insights into the molecular mechanisms involved in mediating the effects of metabolic and immune disturbances in women with PCOS with adverse pregnancy outcomes [67]. One recent endometrial organoid study compared cell-type-specific disease signatures and molecular pathways for PCOS-specific endometrial dysfunction in women with and without PCOS [67]. The investigators examined 248,694 nuclei from six endometrial cell subtypes. They reported a range of differentially expressed genes in cells and pathways related to the processes involved in placentation. Women with PCOS were treated with either metformin or lifestyle management for 16 weeks, followed by repeat endometrial biopsies and the establishment of a second endometrial organoid. Both treatments, either metformin or lifestyle intervention alone, restored multiple differentially expressed genes in each cellular subtype. This study provides new mechanistic insight into PCOS-specific endometrial dysfunction and the potential for reversibility with medical or lifestyle interventions. Future intervention studies are required to demonstrate the effect of dietary modification on clinical outcomes and pathophysiological changes in PCOS, such as IR and inflammation, in human- and laboratory-based research.

Many of the metabolic, endocrine, reproductive, and neuroimmune disturbances that occur in women with PCOS have been shown to be reversed following lifestyle interventions such as the implementation of a balanced diet and exercise [1,16,68]. Diet is a modifiable risk factor, and a healthy balanced diet has been shown to reduce the risk of PE [63]. Pregnant women should eat a diet rich in fruit, vegetables, and whole grains, and healthy sources of fat and protein [56,62]. Women with PCOS should receive lifestyle-oriented counseling and advice, before, during, and after pregnancy, and be considered for inclusion in risk assessment algorithms for the prediction and assessment of PE, as previously discussed.

## 6. Mechanisms of Action of Nutritional Factors in the Pathophysiology of PE

### 6.1. Altered Placental Physiology and the Development of PE

**Figure 1:**
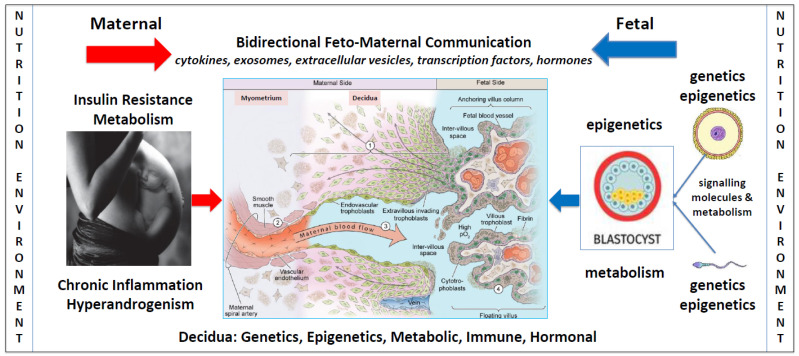
Factors influencing bidirectional fetal–maternal placentation. A schematic model showing the potential impact of nutritional and environmental factors at all stages of pregnancy, including gametogenesis, decidualization, implantation, and placental and fetal development. In normal placental development extravillous cytotrophoblasts proliferate in anchoring columns to successfully invade through the decidua and engage in bidirectional communication with maternal decidual natural killer cells, macrophages, stromal, dendritic, endometrial gland epithelial, and T cells (1). Extravillous cytotrophoblasts transform the distal spiral arteries (2) and these changes mediate high volume flow at low pressure into the intervillous space (3). The blue arrows represent the paternal, maternal, and fetal components. Nutritional and environmental factors influence sperm maturation and development in males [82]. Once sperm enter the reproductive tract, they release signaling molecules that interact with decidual cells prior to fertilization [70]. Human oocytes develop in the mother during embryonic development and are subject to nutritional and environmental factors that influence epigenetic developmental programming [83]. Maternal and paternal nutritional and environmental factors can therefore influence sperm and oocytes prior to fertilization and have the potential to alter bidirectional communication signals during placentation. The red arrows represent the effect of nutritional and environmental factors in maternal pathophysiology and their impact on decidualization, placentation, and embryogenesis. Following fertilization, the zygote and morula receive nutrition from maternal secretions in the fallopian tube [24]. During implantation and throughout the first trimester, both the placenta and embryo obtain nutrition from histotroph fluid that is derived from maternal endometrial gland secretions [24]. These secretions provide glucose, lipids, glycoproteins, and growth factors that stimulate the rapid proliferation of villous trophoblasts, extravillous trophoblast invasion, spiral artery remodeling, and normal development of the placenta [77,84]. At the start of the second trimester, blood enters the intervillous space, resulting in hemotrophic nutritional exchange between the maternal and fetal circulations [85]. Accumulating evidence suggests that pathophysiological changes in women with PCOS, such as insulin resistance, chronic inflammation, and hyperandrogenism, may influence the composition and quality of histotrophic and hemotrophic nutrition, alter bidirectional communication between decidual and placental cells, and effect normal placentation and fetal development. The central diagram in blue is adapted with permission from Kingdom and Drewlo 2011 [86].

Advances in the understanding of normal placental development have paved the way for improved understanding of the pathogenesis and pathophysiology of abnormal placentation in PE [24]. There is a significant overlap between the characteristic features and pathophysiologies of PCOS and PE. PE syndrome may have multiple underlying causes that differ in early- or late-onset presentations, all of which could be influenced by maternal nutritional disturbances [31,69]. Normal development of the placenta involves a complex network of communication signals between fetal-derived trophoblasts and a broad range of maternally derived endometrial cells [25]. The fetal-derived precursor cells of the placenta are affected by sperm-derived signals to endometrial cells prior to fertilization [70,71], paternal and maternal genetics [72], imprinted genes [73], epigenetic reprogramming following fertilization [74], nutritional components of oviduct fluid from cells lining the fallopian tube prior to implantation [24,75], histotrophic nutrition during the first trimester [76], and hemotrophic nutrition following the establishment of significant blood flow into the placental intervillous space [77]. Disturbances of the normal physiology in any of these components could lead to abnormal trophoblast–decidual dialogue and contribute to deficient trophoblast invasion into the uterus and impaired spiral artery remodeling, resulting in placental hypoperfusion and syncytiotrophoblast stress, as is known to occur in PE (Figure 1) [69].

It has been proposed that placental development is independent of, and precedes, embryogenesis, because of a two-way feed-forward dialogue between trophoblast cells and endometrial glands [24]. Hormones from trophoblast cells (human chorionic gonadotrophin, human placental lactogen) and decidual cells (prolactin) stimulate glandular epithelial cells to upregulate the production of nutrients (glucose, lipid droplets, glycoproteins) and growth factors (epidermal growth factors), which in turn feed forward to the trophoblast cells and promote further proliferation and growth of the placenta [24]. This new understanding focuses attention on the role of pre- and post-conception maternal pathophysiology, such as occurs in women with PCOS [3], altered nutrient supply, impaired bidirectional signaling, defective decidualization, and abnormal placentation in PE (Figure 1) [31,78,79,80,81].

### 6.2. Maternal Nutrition, Epigenetics, and Metabolism

Epigenetic processes also play an important role in the development and progression of PE [87], and a wide range of epigenetic changes involving methylation, histone deacetylation, and microRNA have been identified in the placentas of women with PE [87,88]. Epigenetic processes are the mechanism by which environmental influences alter gene expression without changing the structure of DNA. In PE, reduced placental blood flow causes hypoxia and results in epigenetic changes that activate adaptive responses in the placenta and maternal circulation [89,90]. Nutrition, diet, and metabolism regulate epigenetic mechanisms and integrate environmental cues and exposures with cellular responses [91]. Cellular metabolites (acetyl coenzyme A, adenosine triphosphate, nicotinamide adenine dinucleotide) act as nutrient sensors and contribute to the regulation of gene expression via epigenetic mechanisms [92]. Reciprocal crosstalk between epigenetics and metabolism determines molecular programming and cellular function [91]. Maternal obesity can lead to placental dysfunction via chronic inflammation, the dysregulation of metabolic pathways, and epigenetic changes in gene expression [93,94]. Unbalanced diets can alter normal metabolic and epigenetic processes and lead to disturbed cellular function and disease [95]. Recent research in nutritional epigenomics explains how lifestyle interventions (diet and exercise) can restore metabolic and epigenetic homeostasis [96,97]. The interaction between cellular metabolism and the epigenome is a fundamental process in placental development and PE that is influenced by maternal nutritional and environmental exposures [31,87,98,99,100,101,102,103,104,105].

The accumulating molecular, endocrine, metabolic, and epigenetic evidence provides detailed mechanistic explanations for the role of nutritional factors in the pathophysiology of PE that support the previously discussed evidence-based research and recommendations. The following sections provide a brief discussion of the pathophysiological components of PCOS that may be involved in the pathogenesis of altered placental development and function in PE. Studies on the placental effects of maternal pathophysiology are discussed in the following sections and summarized in Table 2.

### 6.3. Insulin Resistance

#### 6.3.1. Insulin Resistance in PCOS

Polycystic ovary syndrome is a life-long problem that is thought to be initiated in utero during developmental programming [106,107]. PCOS has a variety of childhood manifestations and is usually diagnosed in adolescence or early adulthood. Although menstrual disturbance is a common presenting symptom and oligomenorrhea is part of the diagnostic criteria, the underlying pathophysiology can persist after menopause [108]. Although IR is not part of the diagnostic criteria, it is considered intrinsic to the underlying pathophysiology [3,109]. As a result, IR is associated with a number of features of PCOS including symptoms (acne, hirsutism), weight gain, metabolic disorders (diabetes, metabolic syndrome, metabolic-associated hepatic steatosis), hypertension, cardiovascular disease, and pregnancy complications (GDM, PE). Most women diagnosed with PCOS prior to pregnancy present with reduced insulin sensitivity, hyperinsulinemia, and normoglycemia [3,110]. Insulin resistance in early pregnancy has been shown to be predictive for the development of pre-eclampsia [111].

#### 6.3.2. Physiological and Pathological Effects of Insulin and IR

Insulin is a pleiotropic hormone that has multiple cellular and tissue-specific actions, such as the regulation of glucose uptake in some cells (muscle, adipose, vascular endothelium) [112]; increased production of endothelial nitric oxide, resulting in vasodilation in systemic and cardiac blood vessels [113]; reduced excretion of urate [114]; enhanced sodium absorption in the kidney [115]; and multiple metabolic effects [116]. Many of these physiological processes are known to be involved in the pathophysiologies of both PCOS and PE [3,25].

**Table 2:** Studies on placental effects of maternal pathophysiology.

Author (Reference)	Experimental Tissue	Treatment	Main Findings
Hyperinsulinemia			
Nestler [117]	Cytotrophoblasts from human term placenta	Insulin	Inhibition of aromatase via insulin receptor
Vega [28]	Cultured primary first-trimester trophoblasts	Insulin	↑ DNA damage, ↑ apoptosis, ↓ cell survival
O’Tierney-Ginn [118]	Women in early pregnancy	IV GTT	Total insulin secretory response related to placental size and volume
Lassance [119]	Placental villous tissue from TOP	Insulin	Altered transcriptome signature, 30-fold-↓ insulin sensitivity in obese women
Inflammation			
Cotechini [120]	Pregnant rats	Low-dose LPS	Inflammation associated with deficient trophoblast invasion and SA remodeling
Liu [121]	Retrospective case–control study	Endometrial Biopsy	↑ endometrial macrophages, dendritic cells, and T cells, correlated with QUICKI (IR)
Wilson [122]	Primate model of PCOS	Testosterone	Syncytiotrophoblast inflammation
Matteo [123]	Experimental clinical study	Endometrial Biopsy	Abnormal lymphocyte subsets, impaired cytokines (IL-15/18, CL10)
Hyperandrogenemia			
Gopalakrishnan [124]	Pregnant rats	Testosterone	↓ SA remodeling, ↑ placental hypoxia
Sathishkumar [125]	Rat fetus	Testosterone SCI	↓ placental amino acid transport, ↓ placental size and weight
Frolova [126]	In vitro stromal cells In vivo mouse model	DHEA DHEA	Inhibition of decidualization, reduced decidualization
Parsons [127]	Trophoblast cell lines	Testosterone	↑ mitochondrial ROS, ↑ placental oxidative stress
Pan [128]	In vitro cell line	Testosterone	↓ trophoblast cell invasion

Abbreviations: PCOS: polycystic ovary syndrome; IR: insulin resistance; DNA: deoxyribose nucleic acid; ↑: increased; ↓: decreased; TOP: termination of pregnancy; IV: intravenous; GTT: glucose tolerance test; QUICKI: quantitative insulin sensitivity check index; IL: interleukin; CL: chemokine ligand; LPS: lipopolysaccharide; SCI: subcutaneous injection; ROS: reactive oxygen species; DHEA: dehydroepiandrosterone; SA: spiral artery; TG: triglyceride; PE: pre-eclampsia.

Insulin resistance can be defined as an altered cellular response to insulin stimulation that occurs in selective cells and tissues throughout the body [116]. The development of physiological IR is a normal adaptation to pregnancy to ensure adequate placental growth and nutrient supply to the fetus [129]. All women develop progressive IR and hyperinsulinemia throughout pregnancy in response to various hormones (human placental growth hormone, human chorionic gonadotrophin, human placental lactogen) [130,131] and adipokines (adiponectin) produced by the placenta and maternal adipose tissue [132]. Pancreatic insulin secretion can increase by up to 250% during pregnancy to maintain euglycemia [133]. Gestational diabetes is diagnosed if hyperglycemia develops, as defined by national and international reference ranges, and is known to be associated with increased maternal and fetal complications [134,135]. Hyperinsulinemia and/or hyperglycemia related to GDM or PCOS may therefore be involved in pathological placental and fetal responses. Nutritional management is the cornerstone of treatment for both GDM and PCOS [16,136].

Glucose metabolism has been shown to be important for preparation of the endometrium for embryo implantation. Pre-existing IR and hyperinsulinemia have been associated with dysregulated decidualization and are thought to increase the risk of PE [137]. The expression of placental insulin receptors increases with gestational age, along with changes in the tissue distribution [138]. Although insulin-sensitive glucose transporters are expressed in the placenta, the majority are not responsive to insulin stimulation [31]. Glucose transporters on the microvillous membrane of the syncytiotrophoblast move glucose into the cytoplasm via facilitated diffusion down the concentration gradient [77]. As a result, placental glucose transport to the fetus is mostly insulin-independent. This may explain why maternal hyperglycemia results in fetal hyperglycemia and hyperinsulinemia, which can be associated with adverse fetal (macrosomia) and placental effects [136]. Nevertheless, IR can interrupt glucose homeostasis and cause dysfunctional lipid metabolism and excessive inflammation, both of which are associated with PE [31].

#### 6.3.3. Pathological Effects of IR in the Placenta

Pre-existing IR, coupled with the effects of chronic inflammation and hyperandrogenemia, is likely to have additive pathological effects on placental development and may be involved in the pathogenesis of PE [78,129]. Insulin has been shown to inhibit the activity of aromatase in human trophoblasts [117], which may provide a mechanism for connecting hyperinsulinemia with placental androgen excess in women with PCOS. Elevated insulin levels have been found to cause increased DNA damage, increased apoptosis, and decreased cell survival in cultured first-trimester trophoblasts from healthy pregnancies [28]. Transcriptome signatures in placental trophoblasts exposed to insulin showed that the many biological processes (hormonal, cytokine, cell cycle, metabolic) were either up- or downregulated by insulin [119]. Trophoblast cells from the placenta of obese women were 30 times less sensitive to insulin than cells from normal-weight women. The investigators proposed that the enhancement of placental-specific genes supports the concept that insulin promotes both endocrine and growth functions in the placenta, and that IR and obesity can affect the structure and function of the placenta in early pregnancy [119]. Obese women have greater placental lipid accumulation (lipotoxicity and “fatty placenta”) than normal-weight women [139]. Excess placental lipid may be due to changes in fatty acid uptake, decreased fatty acid oxidation, or increased esterification, and may contribute to increased lipid supply to the fetus and fetal adiposity [140]. These data support previous reports that have shown that placental size and volume are strongly related to maternal insulin secretion in early pregnancy [118]. Nevertheless, obesity is often associated with hyperinsulinemia, hyperglycemia, and hypertriglyceridemia, which makes it difficult to separate the effects that may be driven by obesity from those caused by GDM or PCOS in women who have some or all of these problems [140].

#### 6.3.4. Maternal Vascular Endothelial Dysfunction and IR

Maternal metabolism and cardiovascular physiology are altered in pregnancy in response to the increasing demands of placental and fetal growth [141]. Maternal vascular endothelial dysfunction is a classic feature of PE that is thought to be secondary to reduced placental blood flow and the release of pro-inflammatory cytokines, reactive oxygen species, and extracellular vesicles, as well as the imbalance of angiogenic and anti-angiogenic factors [25,142]. Experimental studies have shown that women with PCOS exhibit endothelial dysfunction (impaired endothelium-dependent vasodilation) and reduced responses to the vasodilation effect of insulin [143,144,145,146]. The observed endothelial dysfunction may be related to both elevated androgen levels and IR [143,145]. In addition, PCOS is often associated with chronic hypertriglyceridemia [147], which is a known risk factor for endothelial dysfunction and may cause arteriolar vasoconstriction by altering the regulation of prostaglandins [148]. One systematic review and meta-analysis showed that women that develop PE had elevated serum lipids and triglycerides during all trimesters of pregnancy [80]. Both maternal and placental factors may therefore converge on the maternal endothelium to produce the observed pathological manifestations of PE [78]. Women with PCOS may be at increased risk of PE due to the combined effects of inflammation, hyperandrogenemia, hypertriglyceridemia, and IR on vascular endothelial function.

In summary, accumulating evidence suggests that IR can affect normal placental development and may contribute to the pathophysiology of PE via a variety of mechanisms. Future research involving molecular techniques, computational advances, multiomics data, and endometrial organoids should help our understanding of the effect of maternal IR on placental nutrient and energy utilization, growth pathways, and placental physiology. The investigation of maternal nutritional insults on placental development and function may pave the way for interventions that mitigate the impact on adverse pregnancy-related outcomes, such as those that occur in PE.

#### 6.3.5. Effect of the Oral Contraceptive Pill on IR and Inflammation

The combined oral contraceptive pill (OCP) contains various combinations of estradiol and progesterone that have been found to have a range of adverse metabolic, immune, and vascular effects [149]. In women with PCOS, the combined OCP may worsen IR, particularly if there are other associated metabolic or cardiovascular risk factors [150]. In addition, the OCP can exacerbate the inflammatory state, which may be related to alterations in the renin–angiotensin–aldosterone system (RAAS) [151]. Both inflammatory cytokines and angiotensin II can disrupt insulin signaling and contribute to the pathophysiology of IR [3,152].

It is recognized that it can take months for homeostatic regulatory mechanisms to adjust after stopping the OCP. Symptoms such as acne, weight gain, mood swings, and menstrual disturbance can persist for several months. Alterations in endothelial function may persist for up to 18 months following the cessation of long-term fourth-generation OCP use [153]. It is therefore possible that OCP-related alterations in physiology could have an impact on placentation and pregnancy complications when pregnancy occurs after cessation of the OCP. This may be particularly relevant in women with PCOS, who already have IR and chronic low-grade inflammation.

A recent large prospective cohort study showed that periconceptual OCP use (within 12 months) was associated with an increased risk of preterm birth, low birth weight, and PE (RR: 1.38, 95% CI: 0.99–1.93) [154]. The increased risk of PE was strongest when the OCP was stopped within 3 months of pregnancy. Given that the OCP is the most common medical treatment for PCOS, the relationship between the effects of OCP use and pregnancy complications, such as PE, requires further investigation. Based on the available evidence, women with PCOS should consider ceasing the OCP and using non-hormonal contraception, in addition to the recommended lifestyle changes, at least 12 months prior to trying to conceive.

### 6.4. Chronic Systemic Inflammation

#### 6.4.1. Impact of Maternal Inflammation on Low-Grade Placental Inflammation

Pregnancy is characterized by a state of chronic low-grade inflammation due to the systemic release of a variety of placental cytokines that are required for normal placental development and function [119,155]. PCOS is also characterized by chronic inflammation that is thought to be secondary to poor-quality nutrition, nutritional excess, and other environmental factors that affect metabolic and inflammatory signal transduction pathways [3,8,156]. Women with PCOS also experience a chronic low-grade inflammatory state during pregnancy that has been found to be associated with a higher risk of adverse obstetric outcomes [157,158,159]. Abnormal maternal inflammation has been associated with altered uteroplacental development and function [120], although the precise mechanisms are largely unknown.

Women with PCOS appear to have a pro-inflammatory state that is intrinsic to the underlying pathophysiology [3]. This may contribute to altered endometrial immune cell (natural killer cells, macrophages, T cells) and cytokine profiles (interleukin 15 and 18, chemokine ligand 10), which compromise normal implantation [121,123]. Women with PE have a heightened inflammatory state with elevated pro-inflammatory cytokines and chemokines, both systemically and in the placenta [120,160]. Studies in rodents and non-human primates have provided evidence of mechanistic links between maternal inflammation and PE [120,122]. Lipopolysaccharide-induced inflammation in pregnant rats showed that inflammation was associated with deficient trophoblast invasion and spiral artery remodeling [120]. In addition, inflammation increased the maternal mean arterial pressure and was associated with structural changes in the kidney and proteinuria, as is found in PE [120]. Wilson et al. demonstrated increased syncytiotrophoblast inflammation in a testosterone-induced primate model of PCOS using a novel contrast-enhanced ultrasound technique [122].

Pathological levels of maternal inflammation may result in altered decidual and placental inflammatory responses that affect placental development and function [27,120,161]. Inflammatory processes can contribute to, and exacerbate, the effects of insulin resistance and hyperandrogenism [1]. Previous reviews have discussed the role of chronic low-grade inflammation and altered immune function in PCOS [3] and PE [27].

#### 6.4.2. Interaction of IR, Inflammation, and Hyperandrogenism in the Regulation of Blood Pressure in PCOS and PE

Systemic blood pressure is regulated by a complex network of interdependent physiological mechanisms that work synergistically to ensure adequate tissue perfusion and nutrient and oxygen supply in response to ever-changing environmental conditions [162,163]. This involves a dynamic interplay between metabolic, immune, and neuroendocrine signaling networks that work co-operatively to maintain tissue homeostasis [164]. During pregnancy, additional signaling mechanisms derived from the uteroplacental unit, such as the renin–angiotensin–aldosterone system (RAAS) and angiogenic factors, interact with existing systemic control networks to ensure appropriate fetoplacental blood supply [165]. Pre-eclampsia is characterized by an imbalance of the RAAS and proangiogenic (PlGF, vascular endothelial growth factor) and anti-angiogenic factors (sFlt-1) [166,167]. Women with PCOS can have alterations in their RAAS [168] and vascular endothelial dysfunction [150,169] due to their underlying pathophysiology, which may increase the risk of developing PE in pregnancy.

Several studies have suggested that inflammation and impaired insulin signaling pathways can lead to endothelial dysfunction [145,170]. Endothelial dysfunction has been associated with IR, inflammation, and hyperandrogenism in animal models and in women with PCOS [150,170,171,172]. Insulin can stimulate the expression of nitric oxide synthase (NOS) [173] and endothelin-1 [174] through activation of the mitogen-activated protein kinase pathway. Decreased insulin resistance has been shown to improve endothelial dysfunction in women treated with metformin [150]. Women with PE have been found to have angiogenic imbalance as early as the first trimester of pregnancy [175], and reduced PlGF is used in first-trimester screening tests to predict PE [44]. In summary, women with PCOS may be predisposed to PE and future cardiovascular disease due to the combined effects of IR, inflammation, and hyperandrogenism on endothelial function [143,176].

The RAAS has been found to play a role in the pathophysiology of both PCOS and PE [151,168,177]. Insulin resistance, inflammation, and hormonal factors can all mediate aspects of the RAAS [168,178]. Accumulated evidence has established the role of the innate and adaptive immune systems, and inflammation, in BP regulation [164]. The aldosterone/renin ratio is often increased in women with PCOS and accentuates the inflammatory state [151,168]. Human mononuclear leukocytes express aldosterone receptors [179] that activate inflammatory signaling pathways, and lymphocytes express inflammatory markers following exposure to aldosterone [180]. Immunoregulation of blood pressure is an adaptive survival mechanism that ensures an adequate circulatory response to infectious agents. Evidence suggests that there is an interaction between angiogenic factors and the RAAS that results in upregulation of sFlt-1 in PE [169]. In addition, the insulin signaling system has a reciprocal regulatory role with the RAAS [152,181]. Hyperinsulinemia can disrupt the usual physiological balance of the RAAS by influencing the activity of regulatory enzymes, receptors, and effector proteins [152,182]. Pre-existing dysregulation of the RAAS may predispose women with PCOS to excessive activation of their RAAS in pregnancy and contribute to the pathophysiology of PE [167,176,177].

#### 6.4.3. Impact of Maternal Diet on Placental Inflammation

The mother’s diet during pregnancy can also influence systemic and placental inflammation and may provide a mechanistic link between PCOS and PE [183,184]. Diet quality has been found to influence insulin signaling and inflammatory pathways in rodents and humans [185]. Consuming a higher-quality diet has been shown to improve insulin signaling in the placenta using a mouse model of maternal obesity [185]. Francis et al. examined the effect of consuming a healthy diet on a range of placental proteins involved in metabolic pathways and inflammation [184]. They assessed diet quality using the Healthy Eating Index, which is based on the consumption of vegetables, fruit, dairy, protein, whole grains, and unsaturated fats, with lower intakes of red meat, processed meat, and added sugar [186]. They found that proteins of the p38MAPK inflammatory signaling pathway were lower in the placental villi of pregnant women consuming a healthier diet. Placental p38MAPK is upregulated by pro-inflammatory stimuli and has been linked to placental angiogenesis and further production of pro-inflammatory cytokines [187]. Taken together, these data support the findings of observational studies linking healthy diets to improved pregnancy outcomes and lower rates of PE, as previously discussed.

### 6.5. Hyperandrogenism

#### 6.5.1. Role of Maternal Hyperandrogenemia in the Pathophysiology of PE

The presence of hyperandrogenism in non-pregnant women with PCOS is associated with increased metabolic and cardiovascular risks [188,189,190,191]. Although not all studies report increased adverse pregnancy outcomes in different PCOS phenotypes [192], the majority of published reports demonstrate an association between hyperandrogenism and complications such as gestational diabetes, preterm delivery, and PE [193,194,195]. PCOS is associated with an altered histological structure of the placenta, including microscopic alterations in trophoblast invasion [196,197], which may be increased in women with hyperandrogenism [198]. Maternal hyperandrogenism has also been found to be an independent predictor of PE [195].

The relationship between androgens and maternal cardiovascular and placental function has been investigated in human and animal models [199]. Placental androgen receptor gene expression is increased and placental aromatase mRNA and protein expression are decreased in the placenta of women with PE [125]. Studies using human cell lines have suggested a role for testosterone in mitochondrial dysfunction [127] and the inhibition of trophoblast cell invasion [128]. Blockade of the pentose phosphate pathway using dehydroepiandrosterone was found to inhibit decidualization in endometrial stromal cells and in a mouse model of induced decidualization [126].

Serum testosterone levels of pre-eclamptic women are elevated (two- to three-fold) and are correlated with vascular dysfunction [199]. Elevated androgens in pregnant rats are involved in gestational hypertension (reduced uterine arterial blood flow) [200], endothelial dysfunction (impaired nitric oxide-mediated relaxation in systemic and uterine vessels) [200,201], heightened vasoconstriction in response to angiotensin II [201], decreased spiral artery remodeling (inhibition of angiogenesis, reduced radial and spiral artery diameters, increased UtAPI) [124], placental hypoxia (increased hypoxia-inducible factor levels) [124], and altered nutrient transport (reduced amino acid transport) [125]. All of these factors are known to be involved in the pathophysiology of PE.

#### 6.5.2. Synergistic Effects of IR and Chronic Inflammation on Hyperandrogenemia

Both IR and chronic inflammation can cause and exacerbate hyperandrogenism in women with PCOS [1,3]. Insulin stimulates androgen production in theca cells of normal ovaries and likely contributes to elevated maternal testosterone levels [199,202,203]. Insulin inhibits the aromatase enzyme in human trophoblasts, which may result in a placental contribution to maternal hyperandrogenemia [117]. Chronic inflammation can cause hyperandrogenism via a number of mechanisms, including the disruption of signaling pathways, alteration to epigenetic processes, and post-transcriptional regulatory effects [204]. Hyperandrogenism in PCOS may be due to the synergistic actions of IR and chronic ovarian and systemic inflammation [3,205]. The degree of hyperandrogenism in women with PE varies depending on the sex of the fetus, with higher levels in pregnancies with a male fetus [125]. Therefore, women with PE may have elevated androgen levels due to a combination of fetal, maternal, and placental sources.

A detailed discussion of the effects of hyperandrogenism on maternal vascular and placental functions and its implications for the pathogenesis of PE are beyond the scope of the present report and can be found in previous comprehensive reviews [199,206]. Taken together, these data strongly suggest that many androgen-mediated actions are important contributors to the pathophysiology of PE. The majority of women with PCOS have hyperandrogenemia [207], which may contribute to dysregulated androgen signaling in the placenta and increase the frequency of maternal–fetal complications associated with PE [199].

### 6.6. Role of the Gastrointestinal Microbiome in PCOS and PE

The human microbiome is now appreciated to play a role in many aspects of health and disease [208,209]. Shifts in the gut microbiota are suspected to contribute to both PCOS and PE due to local and peripheral metabolic effects, translocation, and crosstalk with the immune system. Changes in the function of the gut microbiota due to reduced diversity, rather than specific causative organisms, are thought to drive detrimental effects in both PCOS (reviewed in Parker et al., 2022) [210] and PE (reviewed in Colonetti et al., 2023) [211]. In both conditions, reduced alpha diversity (the number of different types of species in a single sample) is thought to be driven by a poor-quality diet and/or obesity, resulting in increased gut permeability, bacterial translocation, systemic inflammation, and metabolic and hormonal disturbances. Alterations in the gut microbiome have also been found to play a role in a wide range of metabolic diseases (type 2 diabetes, metabolic syndrome, metabolic-associated hepatic steatosis) and other diseases associated with PCOS and PE [1].

#### 6.6.1. Summary of the Role of the Microbiome in Women with PCOS

The role of the gut microbiota in the pathogenesis of PCOS was first suggested by Tremellen in 2012 [8]. This theory proposed that consumption of a poor-quality diet caused imbalance of the gut microbiome (dysbiosis), activation of the zonulin pathway, increased intestinal permeability, translocation of lipopolysaccharide (LPS) endotoxin, activation of Toll-like receptors on immune cells, pro-inflammatory cytokine release, chronic low-grade systemic inflammation, impaired insulin signaling, hyperandrogenism, and impaired follicle development. A review of 31 proof-of-concept human studies that investigated components of this specific pathogenic mechanism found that the majority of studies reported reduced alpha diversity and dysbiosis in women with PCOS [210]. Preliminary data showed elevated LPS, LPS-binding protein, and zonulin levels, and a beneficial effect of probiotics and synbiotics. A subsequent review of 18 observational studies found a significant difference in 64 gut microbiota taxa between women with PCOS and controls [212]. A recent meta-analysis of microbiota studies reported that there are specific microbial communities closely associated with PCOS [213]. Since the initial dysbiosis theory was proposed, a range of other microbiome-related mechanisms has been investigated, including choline pathways, primary bile acids, bile acid metabolites, the effects of gastrointestinal hormones, lactate, trimethylamine N-oxide, and short-chain fatty acids (SCFAs) [214,215].

In addition to these observational data, four recent Mendelian randomization studies have investigated a possible causal relationship between the gut microbiome and PCOS. Three studies concluded that there was a causal relationship [212,213,216], and one study did not support a causal relationship [217]. In summary, accumulating evidence supports a role for the gut microbiome in the pathogenesis of PCOS via a variety of possible mechanisms.

#### 6.6.2. Emerging Role of the Microbiome in the Pathophysiology of PE

Although the dysbiotic gut microbiome and PE are consistently associated with one another [211], few studies have attempted to unravel a causal role of these changes. Jin et al. analyzed the fecal microbiome of 92 PE and 86 normal late-pregnancy women and found that SCFA-producing bacteria were depleted in PE relative to normal late-pregnancy women, correlating with a depletion in propionate and butyrate in the placenta [218]. Fecal microbiota transfer of the PE microbiome, but not the late-pregnancy microbiome, was associated with elevated blood pressure, serum interleukin (IL)-17, and LPS levels, and decreased IL-10, goblet cell numbers, and fecal 2-arachidonoylglycerol levels. Taken together, these results indicate that a dysbiotic gut microbiome in PE leads to gut barrier defects, increased systemic LPS, and a reduction in SCFA production. To test if supplementation with SCFA-producing bacteria and SCFAs might alleviate PE, pre-eclamptic rats were treated with *Akkermansia muciniphila* and showed significantly increased levels of butyrate and propionate in stool, serum, and placenta. A concurrent decrease in blood pressure and increased weight and size was observed, although not at the level of the control rats. *A. muciniphila* and propionate treatment were also associated with increased expression of cytokeratin 7 and decreased α-smooth muscle actin expression in the spiral arteries of PE rats, suggestive of improvements in spiral artery remodeling. Treatment with *A. muciniphila* and butyrate was associated with significantly increased levels of claudin-1, occludin, zonulin-1, and goblet cell numbers in pre-eclamptic rat intestines, indicating an improvement in gut barrier function. Furthermore, *A. muciniphila*, butyrate, and propionate in the placental bed were found to increase the proportion of M2 macrophages and decrease the proportion of classically activated macrophages [218]. In another study, the administration of exogenous sodium butyrate in a PE-induced rat model resulted in improved intestinal barrier function, decreased expression of placental inflammatory factors, reduced levels of sFlt-1 and soluble endoglin, and increased PlGF [219]. In addition, both blood pressure and 24-hourly urinary protein levels were significantly reduced in the butyrate-treated rats compared to the controls. These data add to previous research that suggests a role for dysbiosis and SCFAs in PE [220]. Whether or not SCFA-producing bacteria and SCFA supplementation in pre-eclamptic female humans alleviates pathologies and symptoms remains to be tested. Other SCFA-producing bacteria, such as *Bifidobacterium*, could also be tested for their ability to alleviate PE-related pathology and symptoms.

One microbiome study conducted on pre-eclamptic women in East China found an association between PE and the depletion of Bifidobacteria [221]. Another study used two-sample Mendelian randomization analysis to determine a causal relationship between the gut microbiota and PE [222]. Using this meta-analysis approach, they showed that *Bifidobacterium* had a protective effect against PE (OR 0.76, 95% CI: 0.64–0.89, *p* = 8.03 × 10^−4^), while other genera had weaker protective associations. Reverse Mendelian randomization did not associate specific genera with individuals with PE. Xiong et al. also attempted to identify the causal microbiome in PE using a two-sample Mendelian randomization method with false-discovery rate (FDR) correction [223]. Four microbes were identified as being “causal” in PE. *Streptococcus* and the order Enterobacteriales were thought to be protective against PE, and this was supported by Gene Ontology (GO) term enrichment pathway analysis. In contrast, *Akkermansia* and *Olsenella* were thought to be positively correlated with PE risk, even after BMI was ruled out as a factor driving the association with these species. *Olsenella* are associated with endodontic infections [224], suggesting that translocation of the oral microbiome may play a role in PE. Lv et al. observed an expansion in the relative abundance of periodontal disease-associated bacteria, such as *Fusobacterium*, and a decrease in the abundance of SCFA-producing bacteria, such as *Faecalibacterium* and *Akkermansia*, in the gut microbiome of women with early-onset PE [225]. There is ongoing debate regarding the existence and role of a placenta-specific microbiome [226].

In summary, emerging evidence from observational studies, genome-wide association studies, Mendelian randomization studies, and animal studies suggest that the maternal–fetal microbiome (oral, gut, vaginal, placental) may play a role in the pathogenesis and pathophysiology of PE [211,227]. Future studies are required to investigate molecular mechanisms and the role of microbiome-targeted therapeutics such as diet, fiber, probiotics, and SCFAs.

## 7. Identification, Assessment, and Management of Women with PCOS in Pregnancy

### 7.1. Identification of Women with PCOS and Increased Risk of PE in Early Pregnancy

The early identification of women at increased risk of developing PE has been advocated to help reduce the associated maternal and perinatal morbidity and mortality [35,43]. There has been a general lack of awareness of the significant association between PCOS and adverse pregnancy outcomes, including PE. Healthcare professionals do not usually identify women with PCOS during antenatal care, delivery, or the postpartum period [228]. This is reflected in the absence of inclusion of PCOS in risk assessment algorithms for PE, and the lack of specific recommendations for screening, monitoring, and treating women with PCOS during pregnancy [48]. The 2023 international guidelines include a meta-analysis of 109 studies that explored the relationship between PCOS and adverse pregnancy outcomes [53]. As previously discussed, women with PCOS had a significantly increased risk of developing PE in the pooled analysis, which was even greater when only high-quality studies were assessed [42].

Therefore, accumulated evidence from eight systematic reviews over the past 40 years report a significantly increased risk of PE in women with PCOS [42]. In addition, a recent large nation-wide register-based cohort study of 22,947 nulliparous women with PCOS found an increased risk of early-onset (<34 weeks) PE compared to 115,272 non-PCOS controls [229]. Women with PCOS were also more likely to have PE and a small-for-their-gestational-age infant than non-PCOS controls. These data, coupled with the increasing availability of early-pregnancy screening for PE [35], suggest that women with PCOS should be included in risk assessment algorithms and be considered for screening and possible treatment to reduce their risk of developing PE.

### 7.2. Early-Pregnancy Screening and Treatment of High-Risk Women with PCOS

Screening may include measurement of the mean arterial blood pressure, ultrasound with the mean UtAPI, and maternal serum biochemical markers (PAPP-A and/or PlGF) [44,230,231]. Several prospective studies have demonstrated the predictive value of measurements of the serum sFlt-1/PlGF ratio for the diagnosis, monitoring, and management of women at high risk of developing PE [35,43,232,233,234]. Low-dose aspirin treatment starting before 16 weeks and continued to 37 weeks has been shown to significantly reduce the likelihood of preterm pre-eclampsia (62% reduction), preterm birth, and other associated complications [235]. Since women with PCOS may have an increased risk of early-onset PE, prophylactic treatment with aspirin may be particularly beneficial in this patient group.

Only one small study has specifically evaluated the use of low-dose aspirin for treating pregnant women with PCOS. Jamal et al. studied 105 women attending an infertility clinic in 2008 with PCOS diagnosed by Rotterdam criteria [236]. The participants were randomly assigned to three groups of 35 patients. Each patient group took aspirin (one 80 mg tablet daily) or metformin (two tablets twice daily, at 2000 mg), or received no intervention for the duration of their pregnancy. The end-points assessed included UtAPI at 12 weeks’ and 20 weeks’ gestation, and adverse pregnancy outcomes, including PE. Preterm delivery was defined as delivery at less than 37 weeks’ gestation and the definition of PE was not described. The results showed a significant reduction in UtAPI values in the metformin- and aspirin-treated groups at 20 weeks’ gestation, compared to the control group. The aspirin-treated group had a lower but non-significant reduction in UtAPI compared to the women treated with metformin [236]. The lack of a placebo treatment group, non-reporting of early-onset PE, non-reporting of the gestational age upon commencement of aspirin, use of an 80 mg dose of aspirin, and small sample size limits this study’s ability to make conclusions about the effect of aspirin on pregnancy complications. Nevertheless, its finding of a significant reduction in UtAPI at 20 weeks is consistent with results reported in other high-risk groups treated with aspirin [237]. The paucity of studies evaluating the use of aspirin in pregnant women with PCOS may reflect a lack of appreciation for the increased risk of complications and highlights the need for the inclusion of women with PCOS in risk assessment models.

**Figure 2:**
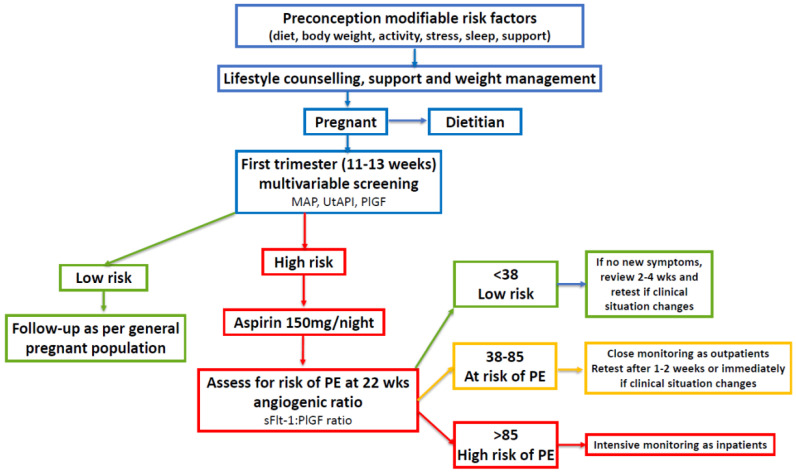
Algorithm for pre-eclampsia screening, lifestyle, and medical management of women with polycystic ovary syndrome in pregnancy. First-trimester multivariable screening is based on references [32,44,231,245]. The use of the sFlt-1:PlGF ratio is based on references [32,35,43,246,247]. Mean arterial pressure (MAP); uterine artery pulsatility index (UtAPI); placental growth factor (PlGF); soluble fms-like tyrosine kinase-1 (sFlt-1).

Low-dose aspirin (<300 mg per day) selectively inactivates endothelial cyclooxygenase and inhibits the biosynthesis of placental thromboxane A [34,238]. The mechanism by which aspirin prevents PE is unknown but may include improvements in placentation, the inhibition of platelet aggregation, and anti-inflammatory effects [238,239,240,241]. Women with PCOS identified as being at high risk during first-trimester screening could be offered low-dose aspirin prophylaxis followed by repeated angiogenic ratio measurements starting at 22 weeks of gestation, as per established protocols (Figure 2) [43].

Many medications, dietary supplements, and herbal treatments have been investigated for the prevention and treatment of PE [242,243]. One systematic evaluation using target product profiles identified 153 candidates for PE. Of the 87 candidates in clinical development, 5 were classified as having high potential for the prevention of PE (esomeprazole, l-arginine, chloroquine, vitamin D, and metformin), and 4 as having medium potential for the prevention of PE (probiotic lactobacilli, dalteparin, selenium, and omega-3 fatty acid) [242]. Calcium supplementation has been shown to reduce the risk of PE in populations with low dietary calcium intake [243]. A recent systematic review identified a number of herbal treatments that were effective in the treatment of PCOS (*Glycyrrhiza glabra* (licorice), *Linum* (chaste berry), *Vitex negundo* (Chinese chaste tree), *Foeniculum vulgare* (fennel), and *Curcuma longa* (turmeric)) [244]. Numerous clinical trials are currently underway to clarify the safety and efficacy of possible future treatments for the prevention and treatment of PE.

### 7.3. Future Investigation of Periconception Low-Dose Aspirin

Low-dose aspirin has been co-administered with a variety of other medications for ovulation induction in women with PCOS-related infertility. These include clomid [248], letrozole [249], tamoxifen [250], and Chinese medicine [251]. When considered together, these studies report improved oocyte quality, higher pregnancy rates, and increased endometrial thickness. It is not possible to determine the impact of aspirin on placentation or pregnancy outcomes from these studies due to the combined administration with other medications. Nevertheless, aspirin is a non-steroidal, anti-inflammatory medication with known hemodynamic and immunomodulatory effects and may have beneficial effects on placentation in high-risk women when initiated in the periconception period. One randomized trial sought to investigate this possibility by comparing low-dose aspirin monotherapy with a placebo treatment [252]: pre-conception aspirin resulted in a non-significant increase in the live birth rate among a large cohort of women with a history of pregnancy loss. A secondary analysis of women who adhered to low-dose aspirin treatment for at least 4 days per week showed improved reproductive outcomes [253]. A further secondary analysis in women with a history of low-grade inflammation, assessed via elevated high-sensitivity C-reactive protein (hsCRP) levels, found that women in the lowest hsCRP tertile had increased live birth rates (RR: 1.35, 95% CI: 1.08–1.67).

Taken together, these studies raise some important issues for the management of women with PCOS, as most women with PCOS have low-grade chronic inflammation and are at increased risk of pregnancy-related complications. Future studies are required to evaluate the impact of periconception aspirin in women with a history of adverse pregnancy outcomes and/or PCOS. In the meantime, the beneficial effects of lifestyle and post-conception aspirin treatment are supported by a significant body of evidence, as previously discussed.

### 7.4. Future Investigation into the Timing of Cessation of Low-Dose Aspirin

Recent studies have suggested that it may be possible to stop aspirin at 28 weeks’ gestation if the angiogenic ratio (sFlt-1:PlGF) and/or the UtAPI are normal [254,255]. Mendoza et al. performed a multicenter, open-label, randomized trial of 936 pregnant women who were identified as being at high risk of preterm PE during first-trimester screening (StopPRE trial) [254]. All women were commenced on aspirin at 150 mg per day before 16 weeks and 6 days of gestation, followed by an sFlt-1:PlGF ratio at 24 to 28 weeks. Participants with a ratio less than 38 were randomized to either continue the aspirin treatment (control group) or discontinue the aspirin treatment (intervention group). The incidence of preterm PE and delivery before 37 weeks of gestation was 1.48% (7/473) in the intervention group and 1.73% (8/463) in the control group (absolute difference −0.25%; 95% CI: −1.86% to 1.36%) [254]. Aspirin treatment that was commenced in these high-risk women before 16 weeks and 6 days and discontinued at 24–28 weeks was not inferior to aspirin continued to 37 weeks for preventing preterm PE in women who had a normal sFlt-1:PlGF ratio at 24–28 weeks. There were no significant differences in any other adverse pregnancy or neonatal outcomes.

Bonacina et al. performed a post hoc analysis of the StopPRE trial described above [255]. In this secondary analysis, women with a UtAPI greater than the 90th percentile were excluded. A total of 836 women were randomized to continue (control group, 416) or discontinue aspirin treatment (intervention group, 420). Preterm PE occurred in 0.7% (3/409) of the intervention group and 1.3% (5/395) of the control group (absolute difference −0.53; 95% CI: −1.91 to 0.85). Discontinuation of the aspirin at 24–28 weeks’ gestation in women with a UtAPI index lower than the 90th percentile was non-inferior to continuing the aspirin treatment until 36 weeks for preventing preterm PE. Women in the intervention group had significantly less minor bleeding complications than women in the control group [255]. These findings are consistent with previous cohort studies showing that low-dose aspirin use during pregnancy may be associated with increased postpartum bleeding and hematoma [256].

The authors of this secondary analysis of the StopPRE trial concluded that high-risk women commenced on aspirin at <16 weeks’ gestation who had either an sFlt-1:PlGF ratio < 38 or a UtAPI lower than the 90th percentile could discontinue aspirin at 24–28 weeks [255]. If further large trials confirm these findings, ceasing aspirin at 24–28 weeks could result in increased compliance and reduced bleeding risk, without the loss of treatment efficacy.

In summary, the diagnosis of PCOS can reliably be made based on a patient’s history upon its first presentation in early pregnancy [257,258]. Women with PCOS should be offered nutritional advice from a dietitian and considered for prophylactic aspirin treatment as per the Fetal Medicine Foundation or local guideline recommendations [230,259]. Women identified as being at high risk during screening should be offered low-dose aspirin (100–150 mg/day, taken at night) starting before 16 weeks of pregnancy, then followed by repeat angiogenic ratio measurements from 22 weeks of gestation (Figure 2) [43,260]. In addition, health practitioners involved in antenatal care should be educated about the risk of pregnancy complications and PE in women with PCOS. Future research should be directed at investigating the underlying pathophysiology that predisposes women with PCOS to an increased risk of PE.

## 8. Strengths and Limitations of the Current Review

### 8.1. Strengths

The current narrative review links high-quality evidence from systematic reviews reporting an increased risk of PE in women with PCOS with evidence from observational studies on the role of lifestyle and nutrition in the pathophysiology of PE. Our summary of the role of the known pathophysiological components of PCOS (insulin resistance, chronic inflammation, hyperandrogenism) with the placental pathophysiology in PE provides an overview of mechanisms described in the scientific literature. We have applied recent developments in screening for PE in early pregnancy with validated second-trimester monitoring using serum biomarkers. This is the first time a combined screening and assessment protocol has been proposed for the management of women with PCOS during pregnancy. Similar recommendations are currently being introduced for women at high risk of pregnancy complications in many parts of the world. Substantial evidence suggests that women with PCOS are at increased risk of PE, and the proposed screening algorithm can be implemented in clinical settings where ultrasound and serum biochemistry testing is available.

### 8.2. Limitations

The present report is a narrative synthesis of many overlapping areas of epidemiology, physiology, and pathophysiology, related to PCOS and PE research. The quality of the presented evidence comes from a variety of sources, including systematic reviews and meta-analyses, observational studies, and laboratory-based scientific research. It is not possible to perform a systematic review or meta-analysis to quantitatively assess the strength of the evidence due to the diverse areas of research that have been linked in the current narrative synthesis. The evidence on the role of nutritional factors in the pathogenesis and pathophysiology of PE comes from observational studies, necessitating cautious interpretation of the findings. Intervention studies currently underway, and developments in organoid research, should provide greater insight into molecular mechanisms in the future. Although all of the components included in the pregnancy assessment and management model have been individually researched and validated in other high-risk populations, they will need to be specifically tested in real-world clinical settings in women with PCOS.

## 9. Conclusions

Polycystic ovary syndrome is a multisystem metabolic and endocrine disorder that is associated with an increased risk of pregnancy-related complications, including PE. International guidelines recommend lifestyle treatment, including the implementation of a balanced diet and exercise, as the first line of treatment for all women with PCOS. A significant body of evidence supports the recommendations of expert advisory groups that healthy diet patterns reduce the risk of PE. PCOS is usually diagnosed in adolescence or early adulthood and presents an ideal opportunity for preventative lifestyle interventions that can be implemented prior to conception to reduce the risk of pregnancy complications. Women with a history of PCOS can also be assessed in early pregnancy and given lifestyle advice and support. In addition, women with PCOS can also be evaluated with first-trimester triple-test screening and advised regarding their eligibility for prophylactic medical therapy to reduce their risk of PE. First-trimester screening can be combined with second-trimester monitoring using angiogenic ratios in high-risk women. This proposed risk assessment and screening algorithm provides the first evidence-based management strategy for the early detection and prevention of PE in women with PCOS. Implementation of the proposed preventative intervention strategy has the potential to reduce pregnancy-related complications and future development of transgenerational chronic disease-related morbidity and mortality in women with PCOS and their offspring. This review highlights the need for further research on pregnancy complications and intervention strategies in women with PCOS.

## Figures and Tables

**Table 1:** Systematic review of studies investigating the risk of pre-eclampsia in women with PCOS.

Author (Year)	Odds Ratio (95% CI) ^1^	Number of Studies	Reference
Boomsma et al. (2006)	3.47 (1.95–6.17)	8	[18]
Kjerulff et al. (2011)	4.23 (2.77–6.46)	12	[19]
Qin et al. (2013)	3.28 (2.06–5.22)	15	[20]
Yu et al. (2016)	2.79 (2.29–3.38)	25	[21]
Khomami (2019)	1.87 (1.55–2.25)	26	[22]
Pan (2021)	2.07 (1.91–2.24)	20	[40]
Riestenberg (2022)	2.03 (1.43–2.87)	15	[41]
Mousa (2023)	2.28 (1.88–2.77)	36	[42]

^1^ CI = confidence interval.

## Data Availability

Not applicable.
